# Exonic splicing signals impose constraints upon the evolution of enzymatic activity

**DOI:** 10.1093/nar/gku240

**Published:** 2014-04-01

**Authors:** Alessia Falanga, Ozren Stojanović, Tina Kiffer-Moreira, Sofia Pinto, José Luis Millán, Kristian Vlahoviček, Marco Baralle

**Affiliations:** 1 Molecular Pathology Group, International Centre for Genetic Engineering and Biotechnology (ICGEB), Padriciano 99, 34149 Trieste, Italy; 2 Bioinformatics Group, Department of Molecular Biology, Division of Biology, Faculty of Science, University of Zagreb, Horvatovac 102a, 10000 Zagreb, Croatia; 3 Sanford Children's Health Research Center, Sanford-Burnham Medical Research Institute, 10901 North Torrey Pines Road, La Jolla, CA 92037, USA; 4 Department of Informatics, University of Oslo, PO Box 1080 Blindern, NO-0316 Oslo, Norway

## Abstract

Exon splicing enhancers (ESEs) overlap with amino acid coding sequences implying a dual evolutionary selective pressure. In this study, we map ESEs in the placental alkaline phosphatase gene (ALPP), absent in the corresponding exon of the ancestral tissue-non-specific alkaline phosphatase gene (ALPL). The ESEs are associated with amino acid differences between the transcripts in an area otherwise conserved. We switched out the ALPP ESEs sequences with the sequence from the related ALPL, introducing the associated amino acid changes. The resulting enzymes, produced by cDNA expression, showed different kinetic characteristics than ALPL and ALPP. In the organism, this enzyme will never be subjected to selection because gene splicing analysis shows exon skipping due to loss of the ESE. Our data prove that ESEs restrict the evolution of enzymatic activity. Thus, suboptimal proteins may exist in scenarios when coding nucleotide changes and consequent amino acid variation cannot be reconciled with the splicing function.

## INTRODUCTION

The rate of protein evolution is traditionally closely linked to the density of protein functional domains (1). However, recent studies highlight the contribution of a myriad of different factors (2). Amongst these, precursor messenger RNA (Pre-mRNA) processing has acquired a role in natural selection (3). This is because the pre-mRNA is now known to harbour important signals for it's processing within the regions containing the coding information. The main recognition sequences (*cis*-acting elements) during the splicing process are: (i) the splice sites that define the exon/intron boundaries, and (ii) the auxiliary splicing regulatory sequences that direct the splicing process. These latter sequences are known according to their location and function as exon splicing enhancers and silencers (ESEs and ESSs, respectively) and intron splicing enhancers and silencers (ISE and ISS, respectively) (4).

Although originally discovered in alternative spliced exons (5), ESEs are now thought to be almost universally present. Indeed, the use of four computationally selected sets of ESE detectors identify potential ESEs in >89% of the exons tested (6). The frequent presence of these elements and their overlapping with coding information raise intriguing implications regarding their roles during evolution. Hypothetically, a non-synonymous nucleotide substitution in the mRNA producing a better performing protein, simultaneously may disrupt an ESE and in this way prevent the correct processing of the exon, ultimately disrupting the production of functional protein (7). Several studies have indeed attested the occurrence of a purifying selection against substitutions in ESEs. A series of hexanucleotide sequences known as RESCUE-ESE, identified as candidate ESEs through computational analysis (8), showed an inverse correlation with single-nucleotide polymorphism (SNP) density in exons, and an increased occurrence in vicinity of exon boundaries (9–12). The need for the preservation of these *cis*-acting sequences for correct exon processing is also evidenced by the finding that at the exon boundaries the synonymous codons are those found with higher propensity in ESEs (13). More generally, a comparative genomics study between human, chimpanzee and macaque has shown a strong tendency for preserving splicing-promoting sequences and a positive selection for their formation (14). All the above computational studies are in accordance with in-depth mutational analysis studies demonstrating that synonymous changes could not evolve freely without potentially compromising proper splicing (15–17).

We hypothesize that the evolutionary constraints ensuring the correct splicing of the Pre-mRNA may be of such extent to even impinge on protein function. In this study, we test this hypothesis experimentally, identifying a scenario where such constraint may have occurred in the human genome during gene duplication.

## MATERIALS AND METHODS

### Construction of minigenes and expression plasmids

Three exon two intron minigenes were created as previously described using the pcDNA 3.1 plasmid as a backbone (Invitrogen) (15). Minigenes for ALPP extended from exons 3–5 whereas minigenes for ALPL extended from exons 4–6. Subsequent mutagenesis of the minigenes was performed standard polymerase chain reaction (PCR) mutagenesis or using a Quick-change site directed methodology (Stratagene).

### Web server

The ESE Analyser web server (EAWS, http://bioinfo.hr/ESE_Analyzer) is based on Perl programming language and uses both the BioPerl modules and Ensembl Perl Application Interface to retrieve data from Ensemble (release 71, April 2012). Pre-collected data for Interpro mode of ESE Analyser are stored in a local MySQL database. The wrappers for the various bioinformatics tools, predicting splicing motifs, including the results parsers are also written in Perl. The web server interface was implemented through Perl CGI modules.

### Prediction of exonic splicing signals

The EAWS web server integrates three approaches: (i) ESEfinder uses position weight matrices of SR proteins which can be downloaded from http://rulai.cshl.edu/cgi-bin/tools/ESE3/esefinder.cgi?process=matrices. The number of detected putative ESEs depend on the threshold set for each matrix, in this study at 90%. (ii) RESCUE-ESE is based on a list of 238 candidate ESE hexamers. To detect RESCUE-ESE hexamers, nucleotide sequences are scanned for the presence or absence of each of them. (iii) ESS were scanned for using the FAS-hex3 set and assigned a score based on the log-odds of frequency in pseudoexons versus exons (18).

### Splice site strength estimation

Strength of exon/intron boundaries were estimated using GeneSplicer program.

### Selection of Ensembl transcripts sharing common protein domains

For each Interpro domain, the list of all genes that contain that domain annotated in Pfam (19) or Smart databases (20) was obtained from Ensembl. For each gene, the list of transcripts together with Interpro annotations was fetched. Only one transcript per gene was selected for the alignment (preferentially transcript annotated in Consensus Coding Sequence database, CCDS) (21), and only one domain instance per transcript, in cases where more than one domain repeats were present in the transcript. In that way, we obtained reliable sets of paralogous sequences at both protein and DNA levels. Those alignments are accessible by selecting Interpro accession number in EAWS. The alignments can be separated into alignments of genes where the domain is constitutively include domain in all transcripts and alignments of genes where the domain is alternatively spliced in the transcripts.

### Splicing assay

Plasmids carrying the minigene were transfected using Effectene (Qiagen) into HeLa cells cultured in Dulbecco's modified Eagle's medium with Glutamax (Invitrogen) supplemented with 10% fetal calf serum (Gibco) according to the manufacturer's instructions. Total RNA was extracted using TRI reagent solution (Ambion) 24 h after transfection and treated with 1 U of RNase-free DNase I (Roche), phenol extracted, EtOH precipitate. Reverse transcription was performed using 1 μg of total RNA with random primers (Promega) and M-MLV reverse transcriptase (Invitrogen). Spliced products were amplified through PCR from the transfected minigene using the primers T7 (5′-taatacgactcactataggg-3′) and SP6 (5′-atttaggtgacactatagaata-3′). PCR products were analysed on a 1.5% agarose gel. Quantification of band intensities was performed in triplicate using the ImageJ64 software.

### Expression and purification of FLAG- tagged enzymes

The expression constructs were transfected into COS-1 using the Effectene transfection reagent (Quiagen). Transfected cells were cultured in OPTI-MEM serum free medium, and conditioned media containing secreted wild-type (WT) and mutant enzymes, were collected 48 h after transfection. Each secreted FLAG-tagged mutant enzyme was purified using anti-FLAG M2 monoclonal antibody affinity column (Sigma, St Louis, MO, USA) as per the manufacturer's instructions.

### Protein quantification and kinetic measurements

The enzyme concentration of each purified sample was determined using a quantitative slot blot assay (22). Briefly, eight 2-fold serial dilutions of FLAG-bacterial alkaline phosphatase (BAP) standard protein (Sigma) and purified recombinant enzymes were prepared in phosphate buffered saline (PBS) in the blocked plates. The FLAG-BAP standard dilutions ranged from 3.5 to 225 ng. The proteins were applied to nitrocellulose membrane through a slot blot apparatus. The quantification of the proteins was then carried out through immunoblotting with the M2 anti-FLAG antibody (Sigma) in a 1.1000 dilution. Quantification of the signal was performed with ImageJ64 Software. Standard curves were obtained by plotting peak area versus known concentration of FLAG-BAP and fitting the data points with linear regression equation. The peak areas of the serially diluted purified protein samples that fell within the detection range of each respective standard curve were used to calculate the protein concentration. The mean value of four peaks from each protein dilution series was used per assay. The standard curve was included in every slot.

Enzyme kinetic determinations were performed using *p*-nitrophenylphosphate as a (pNPP) substrate in 1.0 M diethanolamine (DEA) buffer, pH 9.8, containing 1 mM of MgCl_2_ and 20 μM of ZnCl_2_. For each reaction 8 ng of purified recombinant protein was added to 200-μl of DEA buffer and mixed at 37°C for 1 min with shaking. To calculate *K*_m_ and *V*_max_, substrate concentration was varied between 0.01 and 20 mM. The formation of *p*-nitrophenol was followed as a function of time for 1 h at 37°C and the ?*A*405/min were calculated from the linear part of the time curve. The reaction velocities were determined by measuring the initial slopes of these lines. The initial velocity was plotted against the different concentrations of pNPP and fit non-linear regression to the Michaelis-Menten equation using GraphPad Prism.

### Statistical validation

The *K*_m_ and *V*_max_ values were calculated from six independent experiments and were analysed using R-2.13.1 software. *K*_m_ and *V*_max_ data from WT and mutants enzymes showed normal distribution. The statistical significance was evaluated using Student's *t*-test.

### Comparative genomic analysis and ancestral sequence reconstruction

MultiZ 46-way aligned genomic region relative to the complete Human ALPP gene position hg19:chr2: 233 243 348–233 247 599 was downloaded from the UCSC Genome Browser (23). Subsequent manipulations with the alignment were performed using the custom scripts and rphast package for R 3.0.1 (24). Alignments were trimmed to exon 4 coordinates, flanking 3000 bp in either direction of the exon and filtered to exclude species with no sequence information in the alignment, resulting in a total of 22 aligned vertebrate sequences. Alignment was subjected to the ancestral (HTU) sequence reconstruction using the fastML server (25) with T92 substitution model and maximum likelihood reconstruction method. Ancestral indel threshold was set to 0.9. Tree for reconstruction support was also downloaded from the UCSC Genome Browser and filtered to match sequences in the respective alignment.

Using custom R scripts, sequences from the reconstructed alignments were further trimmed to exon 4 with 30 bp flanks, and then separately scanned for SR protein position weight matrices (90% threshold) and RESCUE-ESE hexamers. Splice site strengths were calculated using the maximum entropy modelling method (26) implemented in R. Alignment was visualized with ggplot2 (27). Phylogenetic tree with colour-coding of splice site strengths and SR protein scores identical to that in the alignment was visualized using the ape package in R (28).

## RESULTS

### Identification of candidate genes

To identify possible candidate genes in which the evolution of exon sequences might be constrained by the presence of ESE elements, we created the computational analysis platform—EAWS (http://bioinfo.hr/ESE_Analyzer) that integrates information from several bioinformatics tools onto an aligned set of paralogous exonic sequences. Specifically, EAWS contains a list of all genes obtained from Ensembl (29) that carry a specific Interpro domain annotation (30). For each Ensembl gene harbouring a particular Interpro domain, a representative transcript is selected and the transcripts are aligned on the coding sequence to match the Interpro domain boundaries. In this way, we obtained reliable sets of paralogous DNA sequences. The web server then integrates the results of computational analysis from several splicing-related tools that predict splice site strengths as well as presence and positions of splicing regulatory elements: Gene Splicer that predicts the 3′ and 5′ splice site strength (31); FAS-ESS that predicts exonic splicing silencers (18); RESCUE-ESE (8) and ESEfinder (32) for the prediction of ESEs.

**Figure 1.**
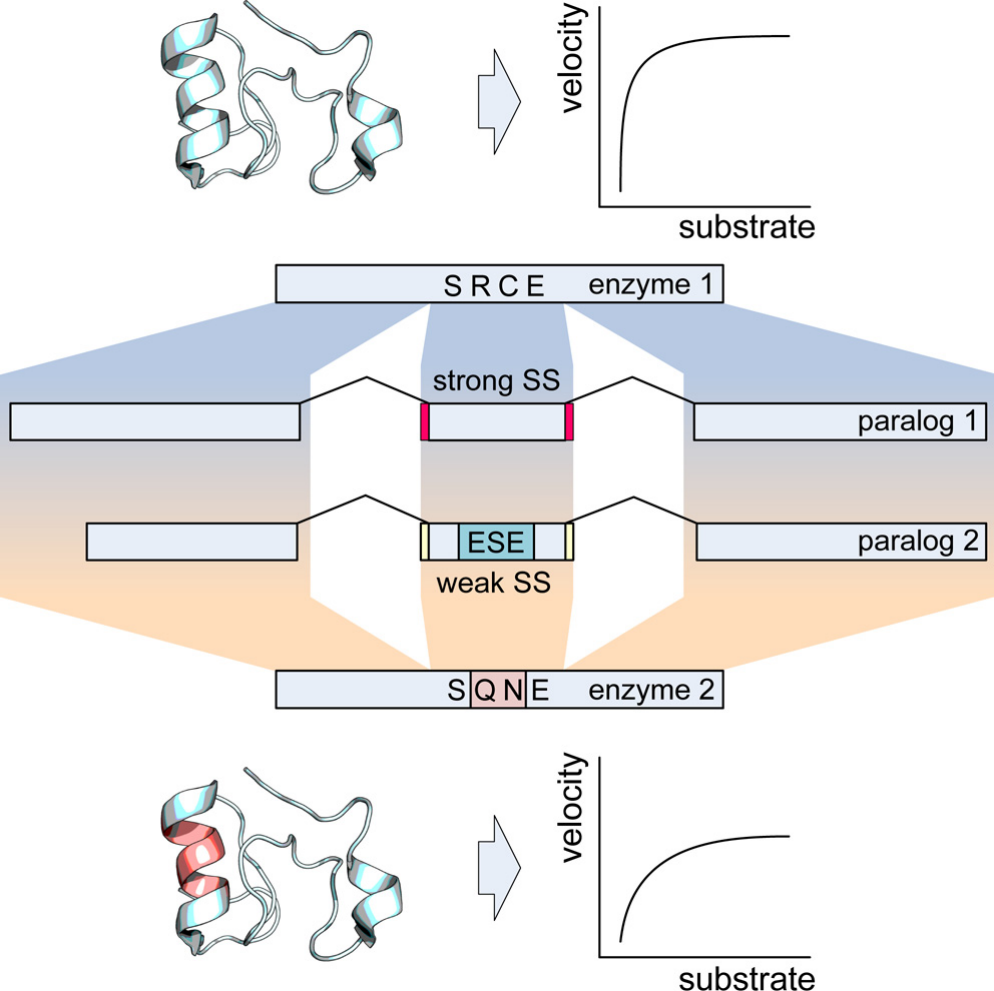
Search criteria for candidate genes. Paralogous protein families were looked for where corresponding exons between family members had different splice site strengths. The exon with suboptimal splice sites should be associated with the prediction of an ESE whose absence in the exon with the strong splice sites is associated with differences in amino acid coding in this region as depicted on top of the exon by one letter amino acid code (in the absence of the ESE, the protein sequence reads SRCE and in the presence SQNE). The family members should also have different kinetic properties.

To simplify analysis of putative sequence constraints imposed by splicing function, the search criteria outlined in Figure 1 was to look at families of human paralogues with conserved exonic organisation and measurable enzymatic activity. The paralogous family members should have different amino acids within or close to the active site and these differences in residue composition would correspond to creation or disruption of an ESE. To aid the screen success rate in identifying authentic ESEs using the bioinformatics tools we also searched for scenarios in which weak/strong splice sites were associated with the presence/absence of ESE within the same paralogous family. This point is in line with the finding that ESEs are essential in exons with non-consensus 3 or 5′ splice sites (33).

**Figure 2.**
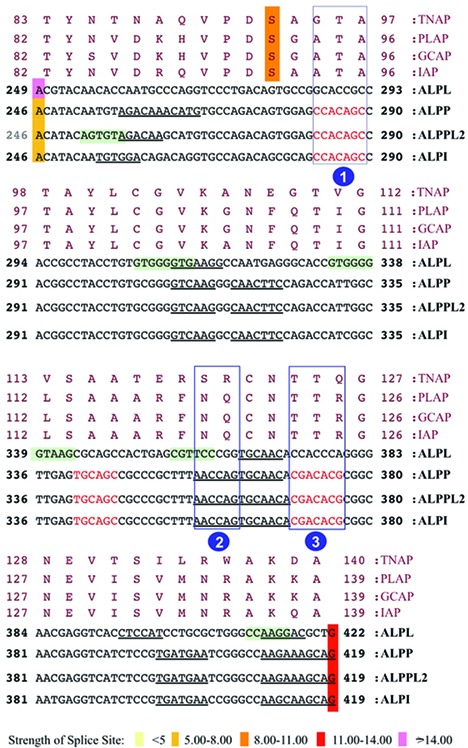
EAWS output of the active site of the human ALP family. The figure shows the alignment of corresponding exonic and amino acid sequences of human APs: placental AP transcript ALPP coding for the PLAP isozyme, the germ cell AP transcript ALPPL2 coding for the GCAP isozyme, the intestinal AP transcript ALPI coding for IAP isozyme and the tissue non-specific AP transcript ALPL coding for the TNAP isozyme. The numbers at the beginning of each block line indicate the amino acid/nucleotide positions in the corresponding proteins/transcripts. Nucleotides in red are predicted ESEfinder motifs, underlined nucleotides are ESE-RESCUE motifs and nucleotides enclosed in a green box are predicted ESS motifs. The ESE areas subsequently studied are boxed (1–3). The active serine of the catalytic domain is highlighted in orange. The exon borders are coloured in different colours depending on the strength of the splice site as calculated by Gene Splicer, with darker colour corresponding to a stronger donor/acceptor.

Several candidate genes were found to comply with our search criteria, the region encoding the active site of the alkaline phosphatase (AP) genes were the most promising. In humans, APs are encoded by four distinct loci, named after the tissues where they are predominantly expressed (34). The expression of placental AP (ALPP, coding for the PLAP isozyme), the germ cell AP (ALPPL2, coding for the GCAP isozyme) and the intestinal AP (ALPI, coding for IAP isozyme) are tissue-specific and the proteins are 90–98% homologous, whereas the tissue-non-specific AP (ALPL, coding for the TNAP isozyme) is approximately 50% homologous to the other three isozymes. Analysis of the APs human transcripts using the EAWS platform (Figure 2) highlighted that the exon encoding a serine involved in the catalytic activity of AP, in particular, the exon 4 of the tissue-specific genes and the exon 5 of the non-tissue-specific isoform had different 3′ splice site strengths. The exon 4 of the tissue-specific AP genes was predicted to have a weak 3′ splice site whereas the corresponding exon 5 of ALPL gene was predicted to have a strong 3′ splice site. The analysis by RESCUE-ESE and ESEfinder identified several ESEs. For the experimental study, we selected three regions with the more significant amino acid differences correlated to weak 3′ splice site of the exon 4 in tissue-specific transcripts and absent in ALPL. ESEfinder highlighted two such hypothetical ESEs, absent in the corresponding sequences of the ALPL exon 5 (Figure 2, boxes 1 and 3), whereas RESCUE-ESE highlighted one (Figure 2, box 2). The nucleotide changes that resulted in the absence of these hypothetical ESEs in ALPL exon 5 are located in a highly homologous region of the gene that is otherwise conserved in the other three tissue-specific family members. It is interesting to note that the analysis for potential ESS did not result in any clear association with the presence of these ESE enforcing the possibility that the ESE and the weak 3′ splice may be interrelated.

### Validation of EAWS prediction

Because the current understanding of the structural properties of human APs come from studies of PLAP and TNAP (35,36), we used the respective genes ALPP and ALPL genes to validate the EAWS predictions.

**Figure 3.**
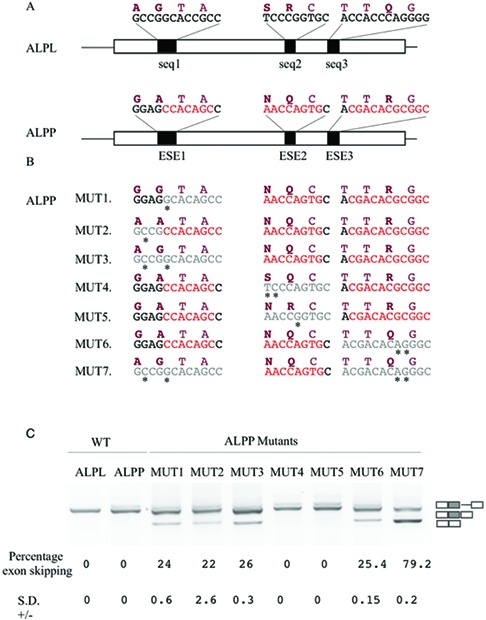
ESE predictions validation. (**A**) Schematic representation of the central part of the WT ALPP and ALPL minigene. Amino acid and nucleotide sequences of the corresponding proteins/transcripts under validation for the presence of ESE are shown above each exon. Nucleotides in red are predicted ESE motifs and amino acids in bold are those that are different between the two transcripts. (**B**) Sequence changes performed to create the constructs MUT 1–7 on the ALPP WT minigene in the regions of the first, second and third ESEs are highlighted with an asterisk and the areas further emphasised in grey. The corresponding amino acid change that this would cause is also highlighted on top of the nucleotide sequence. (**C**) The splicing pattern observed after transfection of these constructs in HeLa cells. On the right hand site of the gel a schematic representation of the splicing product obtained can be observed. The percentage of skipping and standard deviation (SD) are indicated below each lane and represent the mean of three experiments. A minimal amount of cryptic intron inclusion, an artefact of the minigene system is also observed.

The nucleotide sequences encompassing ESE 1 (Figure 2, box 1) in ALPP contained a non-synonymous difference compared with the corresponding sequence in ALPL (Figure 3A. ESE 1 compared to seq 1). To test the prediction of EAWS that the nucleotides coding for the amino acid at this position, alanine 94, was a part of an ESE element in ALPP and that the corresponding nucleotides coding for glycine in ALPL were not, we created a 3 exon 2 intron minigene (15) in which the central exon was the one under study (ALPP exon 4). The triplet encoding for the amino acid difference was then altered so as to be identical to its counterpart in ALPL exon 5 (Figure 3B, MUT 1). As the glycine immediately upstream of the alanine 94 in ALPP also differs to that in ALPP, not withstanding it was not identified as an ESE, we also created a minigene where the nucleotides encoding glycine 93 were exchanged for their counterparts in ALPL that coded for an alanine (Figure 3B, MUT 2). Analysis of the mRNA processing of these minigenes following transfection of the constructs in HeLa cells showed that the nucleotides associated with both the amino acid differences between ALPP and ALPL adjacent to and within ESE 1 were part of an ESE in ALPP that was not present in ALPL as these substitutions resulted in partial exon skipping (Figure 3C, compare MUT 1 and MUT 2 with WT ALPP). A minor amount of cryptic intron inclusion, an artefact of the minigene system was also observed in the ALPP wild-type minigene. Concurrent substitution of both codons in ALPP for those of ALPL (Figure 3B, MUT 3) resulted in equivalent levels of exon skipping as observed with the single substitutions of the nucleotides of each codon, indicating that these nucleotides more than likely form a single ESE element (Figure 3C, MUT 3).

The putative ESE region 2 encloses two amino acid differences, between ALPP and the equivalent region in ALPL (Figure 2, box 2 and Figure 3A; ESE 2 compared with seq 2). mRNA processing of hybrid minigenes in which the nucleotides for these codons were mutated into corresponding triplets in ALPL (Figure 3B, MUT 4 and MUT 5) did not result in any differences in splicing pattern from the wild-type minigene indicating that these nucleotides did not code for an ESE (Figure 3C, MUT4 and MUT5). On the other hand, a minigene in which the nucleotides corresponding to the third putative ESE that coded for arginine 125 (Figure 2, box 3 and Figure 3A, ESE 3 compared with seq 3) where exchanged for those in ALPL (Figure 3B, MUT 6) exon skipping was observed indicative that these were part of a functional ESE element (Figure 3C, MUT 7).

To investigate if the two ESEs were acting in a synergistic manner we created a minigene in which the nucleotides identified to be coding for ESEs (Gly93 and Ala94 in ESE 1 and Arg125 in ESE 3) were exchanged for the corresponding nucleotides in ALPL (Figure 3B, MUT 7). Transfection into HeLa cells, followed by RT-RCR analysis, resulted in a significant increase of exon skipping (79.2%) compared with that observed when either ESE element regions were substituted for the corresponding region of ALPL, demonstrating a cumulative effect of ESE sequences present in these two areas (Figure 3C).

### The ESEs in ALPP compensate a weak 3′ splice site

**Figure 4.**
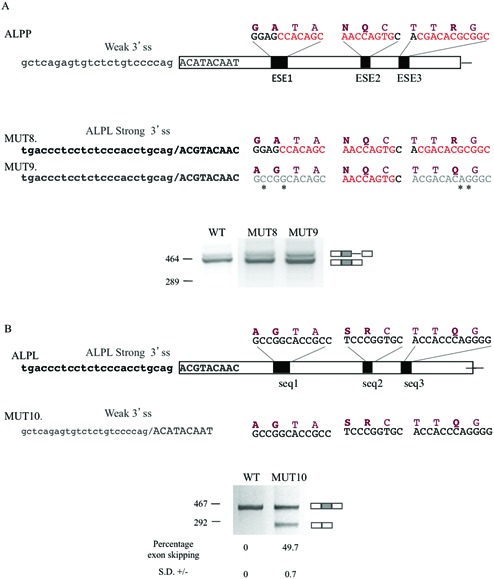
Interplay of 3′ splice site strength and ESEs. (**A**) Schematic representation of ESE (nucleotides in red) were predicted shown above the exon as well as the 3′ splice site at the start of the exon. The mutants, MUT 8 and MUT 9, both have the 3′ splice site of ALPP substituted from the stronger one of ALPL, however, in the later the first and third hypothetical ESE sequences, which were shown to be functional ESE, are mutated in such a way as to disrupt them (mutated nucleotides are marked with an asterisk). Lower panel, splicing pattern observed upon transfection of these constructs in HeLa cells. (**B**) Schematic representation of the central part of the WT ALPL minigene with the proteins/transcripts region above the exon that corresponds to that of the ESE in ALPP as well as the 3′ splice site at the start of the exon. MUT 10 carried the weak 3′ splice site of ALPP. Lower panel, splicing pattern observed upon transfection of these constructs in HeLa cells. On the right hand site of the gels, a schematic representation of the splicing product obtained can be observed. The percentage of skipping and standard deviation (SD) are indicated below each lane and represent the mean of three experiments.

A compensatory relationship has been shown to exist between splice sites strength (agreement to splice site consensus) and ESEs (37). Bearing in mind that ALPL has the same characteristics as the ancestral copy of the gene from which this family is derived (38) and as the predication of the 3′ splice site strength of the ALPP exon 4 was suboptimal compared with the ALPL exon 5 3′ splice site (Figure 2), we investigated if this sort of compensation may have occurred in the two paralogous genes during evolution. We therefore prepared minigenes where the 3′ splice site of ALPP was switched for that of ALPL in the wild-type minigene as well as in the ALPP minigene in which both ESE elements previously mapped were disrupted (Figure 4A). Analysis of the mRNA processing of these shows that this remains unaltered compared with the wild-type minigene, even in the absence of the two ESEs previously shown to result in more than 70% of exon skipping (compare Figure 4A, MUT 9 to Figure 3C, MUT 7). Thus, improvement of the 3′ splice site strength eliminates the need for ESE in this exon. Conversely, the insertion of the ALPP 3′ splice site into the ALPL minigene results in partial exon 5 skipping that does not occur with the wild-type ALPL minigene, indicating the need for ESEs in the presence of the suboptimal ALPP 3′ splice site (Figure 4B).

### Kinetics properties of PLAP mutants

To assess if the requirement for a functional ESE can affect protein function and constrain the nature of the coded amino acids hybrid proteins were created in which the amino acids, coded by ESE motifs in PLAP were replaced with those in the corresponding region of TNAP and the protein activity analysed. The recombinant enzymes were produced through cDNA expression and engineered to be secreted by replacing the peptide sequence of ALPs glycosylphosphatidylinositol anchoring signal with the FLAG octapeptide (DYKDDDDK), generating secreted-tagged enzymes upon transfection (39).

The PLAP-FLAG first ESE protein, where the amino acids encoded within the ESE 1 region in PLAP were exchanged with the corresponding residues in TNAP (Gly93 > Ala and Ala94 > Gly), had a significantly lower *K*_m_ (*P* < 0.01) in respect to WT PLAP-FLAG. The decrease in *K*_m_ was not accompanied by any significant variation in *V*_max_ and consequently in *k*_cat_ between the two enzymes (Table 1). Exchanging the amino acids in ESE 3 between PLAP and TNAP (Arg125 > Gln) was found to result in a protein with a *V*_max_, and consequently *k*_cat_ significantly increased with respect to WT PLAP-FLAG (*P* < 0.001) whereas no significant variation in *K*_m_ was observed (Table 1).

**Table 1.** Kinetic parameters of wild-type and mutant TNAP and PLAP enzymes

Enzyme	*K*_m_ (mM)	*V*_max_ (μM s^−1^)	*K*_cat_ (s^−1^)	*K*_cat_/*K*_m_ (s^−1^ μM^−1^)
WT PLAP-FLAG	0.2239 ± 0.017	0,0751 ± 0,001	240.4 ± 0.001	1.073
WT TNAP-FLAG	0.4365 ± 0.051	0,1719 ± 0,005	573 ± 0.003	1.312
PLAP-FLAG first ESE (G93A;A94G)	0.1519 ± 0.015	0,0663 ± 0,001	212.4 ± 0.001	1.398
PLAP-FLAG third ESE (R125Q)	0.2135 ± 0.009	0,1030 ± 0,001	329.6 ± 0.002	1.543
PLAP-FLAG first ESE (G93A;A94G)/third ESE/(R125Q)	U	U	U	U

Measurements were performed at pH 8.9 using pNPP as substrate in the presence of 1 mM of MgCl_2_ and 20 μM of ZnCl_2_. U, undetectable.

A third recombinant protein was produced, PLAP-FLAG first/third ESE, to analyse the possible effect of all the three amino acid differences associated with the nucleotide sequence corresponding to the ESE in PLAP and not TNAP on the enzymatic activity. The three substitutions resulted in an enzyme that did not have any activity over background levels of the assay. This is most likely due to incompatibility of these changes with the other amino acid variations present in the whole structure of TNAP and PLAP.

## DISCUSSION

Exonic sequences, such as exons 9 and 12 of the Cystic fibrosis transmembrane conductance regulator and Fibronectin EDA have been shown to be constrained from an evolutionary viewpoint by splicing requirements. This type of constraint has been hypothesized to consequently restrict functionally productive genome variability (16,40–42). Using the alkaline phosphatase gene (ALPP) family as a model we have now performed a detailed analysis of the dual role of coding sequences—amino acid coding and ESE conservation specifically analysed in terms of the effect on protein performance.

Current views of the evolutionary history of ALP propose that the ancestral gene coded for a tissue-non-specific alkaline phosphatase and that subsequently, through a process of gene duplication and additional mutations, the intermediate intestinal gene arises from the tissue-non-specific precursor gene (ALPL like). This gene, through different alterations in the promoter regions of the genes (43,44), became the progenitor of intestinal, germ cell and placental genes (45). In fact, the ALPP gene represents a late evolutionary event since enzymes with the properties of PLAP (protein product of ALPP gene) were found expressed only in placenta of chimpanzee, orangutan and human (45,46), while in several other mammalian species PLAP and TNAP (protein product of ALPL gene) are equivalent (34,47). The human alkaline phosphatase paralogous gene family is thus an example of subfunctionalisation because these genes are expressed in different tissues still preserving the same features of the catalytic mechanism (48), but with different enzymatic characteristics. These property differences may reflect specific in vivo functions (34). Indeed, the amino acid variations between the tissue-specific ALPP and those in the corresponding region of ALPL, including the Gly93, Ala94 and Arg125, may have evolved due to protein variations associated with enzyme specialisation or acquisition of novel protein properties. An alternative hypothesis that we favour is that they reflect an evolutionary pressure linked to spicing restrictions. In this study, we have demonstrated that the nucleotides coding for these amino acids specify for ESEs necessary for the correct inclusion of the exon 4 in the mRNA due to its weak 3′ splice site. These nucleotides are different from those in the corresponding region of the ALPL in a region otherwise highly conserved, where the ESEs were shown to be absent and unnecessary due to the strong 3′ splice site of this exon. The different nucleotide sequences in turn code for different amino acids that, we also demonstrated, when placed into ALPL change the enzymatic characteristics of the protein. The fact that these amino acids in ALPP could not be altered to those of ALPL without aberrant RNA splicing and negative consequences on the inclusion of the exon suggests the existence of splicing constraints on protein sequence evolution. As ALPL is considered the ancestral copy of AP (38) at least two hypotheses are possible to explain the temporal succession for the differences in splicing regulatory motifs: (i) Mutations in the exon may have led to creation of one of the ESE nucleotide sequences and consequent variations in the amino acid sequence. Subsequently nucleotide changes could occur either in the exonic region generating the second ESE or within the 3′ splice site. In the former case, it would have no effect on splicing but the outcome would only be on protein activity. If, on the other hand, it were to occur in the 3′ splice site reducing its strength, with just one ESE, a partial aberrant splicing could occur as we have shown in Figure 3, however, enough correctly spliced mRNA ensures maintenance of the AP function. (ii) The initial mutation weakens the 3' splice site, this case partially overlaps with the sequence ESE creation then 3′ splice site mutation of hypothesis 1. In both there is minimal splicing of correct mRNA ensuring protein function (Figure 3), then optimisation of mRNA and protein production could be achieved selecting mutations that create an ESE, the price to pay for this optimisation could be amino acid variations that change the protein characteristics.

To attempt to determine the temporal succession of events that resulted in this constraint we carried out a comparative genomic alignment of ALPP orthologues and reconstructed ancestral sequences on which ESE-rescue motif and 3′ splice site strength analysis were performed (Supplementary Figure S1). This analysis could not discriminate conclusively between the possibilities that the ESEs appeared before or after the weakening of the 3′ splice site. An analysis of the phylogenetic relationships of the orthologues along with reconstructed ancestral nodes, highlights that the nodes prior to the branch leading to the hypothetical taxonomic units carry a strong 3′ splice site and do not have the ESE elements we characterized until the node leading to primates (Supplementary Figure S2). Considering ALPL is the more ancestral like of the AP family (38), we speculate that a mutation in the splice site occurred first, weakening the 3′ splice site, and thus exerting a selection pressure towards stronger splicing enhancers to compensate for the proper exon inclusion. This assumption is compatible with our data for the ALP family as in this case we have established that in the presence of the weak 3′ splice site some exon inclusion is still occurring (Figures 3 and 4). This hypothesis is also in line with the splicing compensation model (14) in which selection for splicing-positive mutations takes place to counter the effect of an ongoing splicing-negative mutational process, with the exon as a whole being conserved. This model paints a picture of exon evolution as a dynamic interplay between helpful and harmful mutations both at enzymatic function and Pre-mRNA splicing level, continuously at work.

As ALPP is 90% homologous to the other two tissue-specific isozymes it is tempting to hypothesize that the same results obtained in ALPP in regards to splicing and protein activity would occur also in ALPPL2 and ALPI, making it possible to extend our model to a more general evolutionary theory of splicing motifs from the tissue-non-specific ancestral gene to the less distant tissue-specific paralogues. In this case, however, it is interesting to note that due to the degeneracy of the genetic code scenarios will also exist similar to that possibly occurring in ALPI, where the nucleotides are still able to code for the amino acid observed in ALPL (glycine) while still maintaining the ESE (Figure 2).

Irrespective of how the genes evolved, the implication of the double purpose of the coding sequence on gene evolution is clear. Sequences need to comply with two possibly conflicting functions: the requirement to maintain the exon included in the mature mRNA and the selection of sequence variants that encode amino acids altering enzyme performance. With the splicing-driven selective pressure preceding the protein function selection as a precondition for its translation (7), we can conclude that the protein activity may not be as optimal as possible. Indeed, our results for the first time show that genomic variations coding for amino acidic substitution may be constrained at splicing level first in order to be included in the messenger and only then confer particular characteristics to protein performance.

## SUPPLEMENTARY DATA

Supplementary Data are available at NAR Online.

SUPPLEMENTARY DATA
