# Supplementary material for: Exonic splicing signals impose constraints upon the evolution of enzymatic activity
Source: Nucleic Acids Res. 2014 Apr 1;42(9):5790–8. doi: 10.1093/nar/gku240 (PMC4027185; doi:10.1093/nar/gku240)
Supplement: SUPPLEMENTARY DATA [file supp_42_9_5790__index.html]

Exonic splicing signals impose constraints upon the evolution of enzymatic activity — Exonic splicing signals impose constraints upon the evolution of enzymatic activity — SUPPLEMENTARY DATA 

# Exonic splicing signals impose constraints upon the evolution of enzymatic activity

## SUPPLEMENTARY DATA

**Files in this Data Supplement:**

- SUPPLEMENTARY DATA
