# Supplementary material for: Exonic splicing signals impose constraints upon the evolution of enzymatic activity
Source: Nucleic Acids Res. 2014 Apr 1;42(9):5790–8. doi: 10.1093/nar/gku240 (PMC4027185; doi:10.1093/nar/gku240)
Supplement: SUPPLEMENTARY DATA [file supp_gku240_nar-03269-a-2013-File006.pdf]

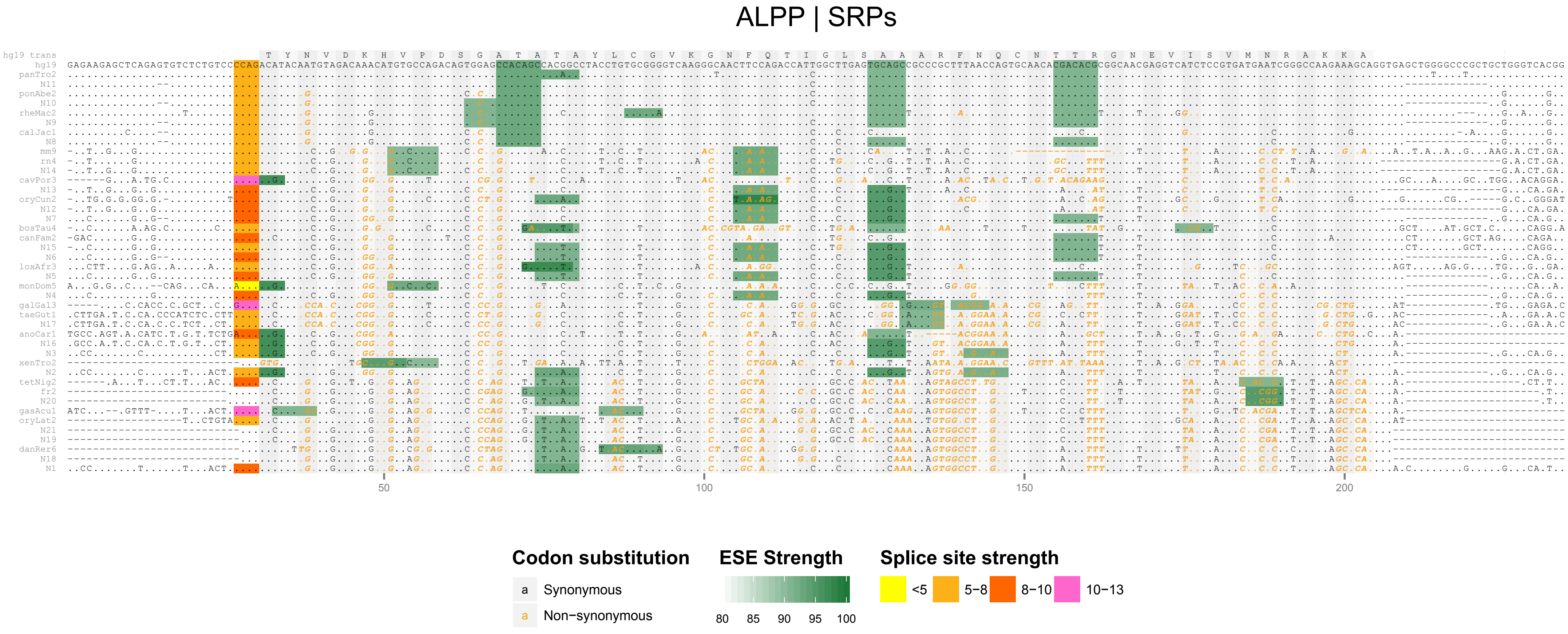

Supplementary figure 1

Comparative genomic alignment of ALPP orthologues along with reconstructed ancestral nodes (see Supplementary figure 2). 3' splice site strength is colour coded acording to the splice site strength (the stronger the splice site the darker the colour). ESEfinder motif with more than 90% consensus are highlighted in green. The intensity of the shade of green reflects the strength of the score of the motifs. Reconstructed ancestral sequences (hypothetical taxonomy unit nodes) are named N1-N21 while the genomic sequence names follows the UCSC convention (http://genome.ucsc.edu/FAQ/FAQreleases.html).

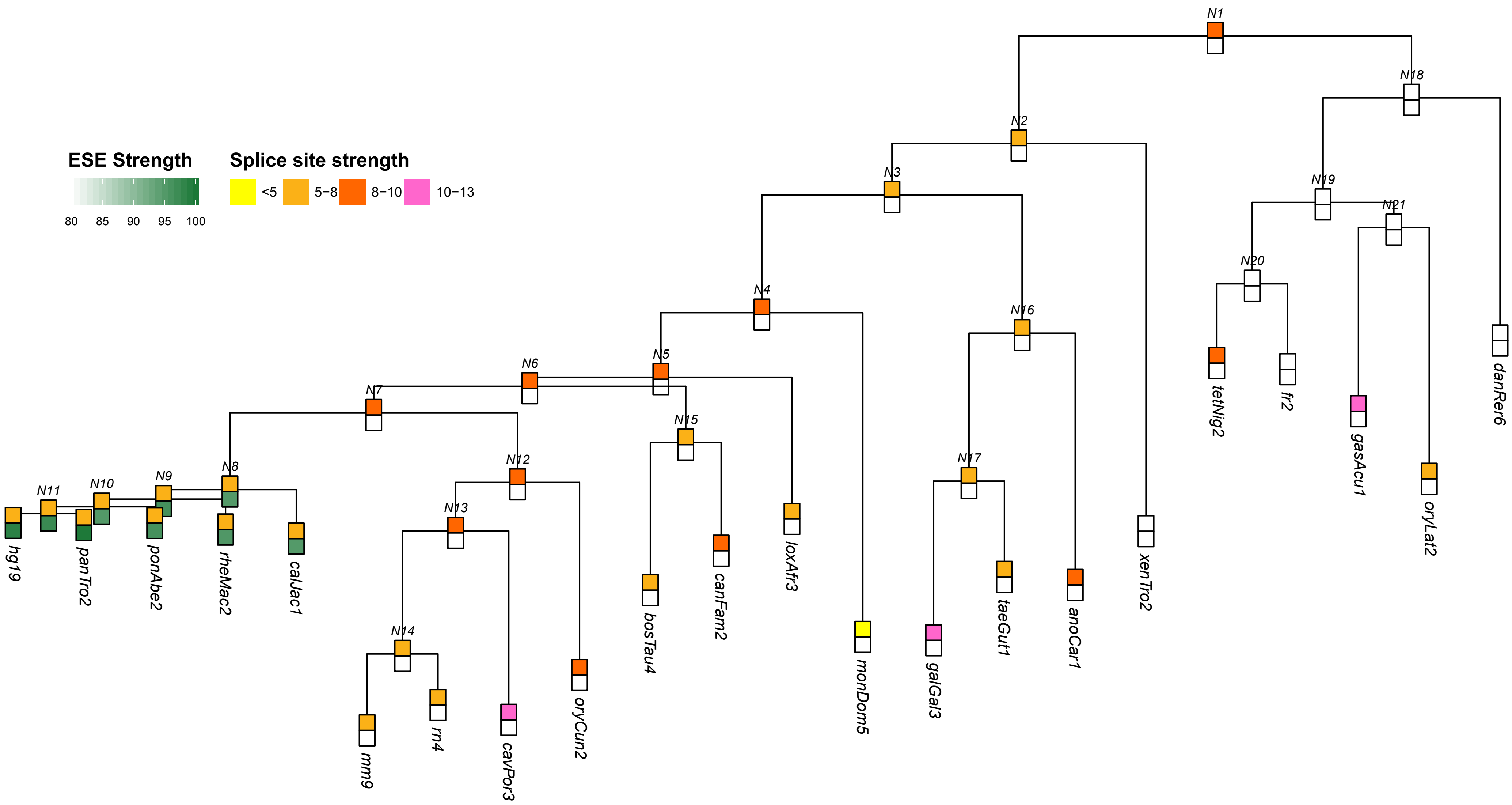

Supplementary figure 2
Phylogenetic tree of the ALPP orthologues and reconstructed ancestral sequences. Boxes represent the presence/absence of an ESE in investigated positions relative to the strength of 3’ splice site.
